# Antioxidant Resveratrol Increases Lipolytic and Reduces Lipogenic Gene Expression under In Vitro Heat Stress Conditions in Dedifferentiated Adipocyte-Derived Progeny Cells from Dairy Cows

**DOI:** 10.3390/antiox10060905

**Published:** 2021-06-03

**Authors:** Gitit Kra, Jayasimha Rayalu Daddam, Hadar Gabay, Sara Yosefi, Maya Zachut

**Affiliations:** 1 Volcani Center, Department of Ruminant Science, Institute of Animal Sciences, Agriculture Research Organization, Rishon Lezion 7505101, Israel; gititk@volcani.agri.gov.il (G.K.); jayasimha@volcani.agri.gov.il (J.R.D.); hadar672@gmail.com (H.G.); 2 Department of Animal Science, The Robert H. Smith Faculty of Agriculture, Food and Environment, The Hebrew University of Jerusalem, Rehovot 76100, Israel; 3 Volcani Center, Department of Poultry Science, Institute of Animal Sciences, Agriculture Research Organization, Rishon Lezion 7505101, Israel; yosefis@volcani.agri.gov.il

**Keywords:** antioxidant, DFAT cells, docking, *FASN*, heat stress, resveratrol, reactive oxygen species

## Abstract

Heat stress (HS) induces oxidative stress by increasing reactive oxygen species (ROS), and the polyphenol resveratrol (RSV) has been shown to have antioxidant properties by reducing ROS. Hence, we aimed to examine the effects of RSV, HS and their interaction on bovine adipocytes. We generated bovine dedifferentiated adipocyte-derived progeny (DFAT) cells from subcutaneous adipose tissue and examined the effects of RSV (100 µM), heat conditions: isothermal (ISO-37 °C), short heat (SH-41.2 °C for 1 h) and long HS (LH-41.2 °C for 16 h), and their interaction on gene expression in DFAT-cells. In medium of DFAT-cells treated with RSV, malondialdehyde levels were reduced and oxygen-radical absorbance-capacity levels were increased compared to control. Treating DFAT-cells with RSV increased the relative mRNA expression of stress-induced-phosphoprotein-1 (*STIP1*) and the expression of hormone-sensitive-lipase (*LIPE*) and perilipin-1 (*PLIN1*), whereas it reduced the expressions of fatty-acid-synthase (*FASN*) and of pro-inflammatory chemotactic-C-C-motif-chemokine-ligand-2 (*CCL2*) also under HS. Moreover, reduced protein abundance of *FASN* was found in RSV-treated DFAT-cells compared to controls. Molecular docking of RSV with *FASN* confirmed its possible binding to *FASN* active site. This work demonstrates that RSV has an antioxidant effect on bovine DFAT cells and may induce adipose lipolysis and reduce lipogenesis also under in vitro HS conditions.

## 1. Introduction

In dairy cows, environmental heat stress (HS) leads to decreased productive and reproductive functions [1,2]. Environmental HS caused by high ambient temperature and humidity conditions induces NF-E2-related factor2 (NRF2) mediated oxidative stress pathway in adipose tissue of late pregnancy dairy cows by affecting several biomarkers and increasing reactive oxygen species (ROS) along with lipid peroxides [3,4,5]. Due to oxidative stress, dysfunctional responses of immune and inflammatory consequences occurs, which may be a nexus between infection and health disorders in dairy cows [6,7,8,9]. The imbalance of ROS can be improved by the complex antioxidant system, maintaining redox balance in cells. In this antioxidant defense system, different types of antioxidant enzymes and non-enzymatic low molecular weight antioxidants play an important role [10]. HS causes interruption in these enzyme expressions and functions and accumulates superoxide anion radicals. One dietary strategy to enhance dairy cows’ health is by supplementation of antioxidants in the nutrition, which can protect from oxidative stress. Antioxidants can scavenge free radicals by inhibiting the initiation steps leading to the termination of lipid peroxidation process [11]. In the last few years, several studies have used plant polyphenols as antioxidants to reduce the oxidative stress effects in dairy cows [12]. Yet, polyphenol supplementation is still an emerging area in the nutrition of dairy cattle. Recent research also showed different effects of polyphenols in bovine derived cells [13,14].

Resveratrol (trans 3, 5, 4′-trihydroxystilbene; RSV) is a non-flavonoid polyphenol found in a variety of plant species, like grapes [15], rhubarb [16], blueberries [17], peanuts [18] and others. This compound has many biological activities like antioxidative [19], anti-inflammatory and immunomodulatory properties [20,21], anticancer [22,23], estrogenic effect [24], anti-neurodegenerative [25] and antimicrobial activity [26,27]. One of the mechanisms of RSV is connected with activation of sirtuin 1 and phosphorylation of adenosine 5-monophosphate-activated protein kinase (AMPK) pathway. The activated AMPK downregulates Acetyl-CoA carboxylase and this inhibits lipogenesis resulting in increased energy metabolism [28].

In bovine primary adipocytes, HS directly increases lipolytic response, which is through increased PKA mediated phosphorylation of the lipolytic enzyme hormone sensitive lipase (HSL also known as LIPE) and perilipin-1 [29]. In recent years, discovery of adipose tissue as a complex active metabolic and endocrine organ has been given considerable attention in research [30,31]. Therefore, for better understanding of the HS effect on biological mechanisms in adipose tissue and its role in the physiology of the dairy cow could contribute to developing novel dietary approaches towards optimizing performance of dairy cows during the summer. We hypothesized that RSV may exert an antioxidative effect on bovine adipocytes thus partly alleviating the negative impact of HS. However, the RSV effects on energy metabolism of adipose tissue are not clear. Therefore, in this work we examined the effects of RSV on bovine de-differentiated adipocyte-derived progeny cells (DFAT) under short and long in vitro HS conditions. We demonstrated the direct effect of RSV on the gene expressions related to lipolysis, oxidative stress, inflammation and lipogenesis in isolated bovine DFAT cells in vitro. In addition, we examined the effects of short and long HS, and the combination of RSV and heat treatments on different gene expressions in DFAT cells, as well as the effect on protein abundance of *FASN* and used computational methods of molecular docking to find possible interaction of RSV with *FASN*, which could affect lipogenesis in adipose tissue.

## 2. Materials and Methods

### 2.1. Animal and Tissue Samples

Subcutaneous adipose tissue samples (approximately 500 g) were harvested from the hind leg area of Holstein Friesian cows (*n* = 5) at an abattoir (Haifa, Israel) during the winter season. Adipose samples were trimmed and washed with prewarmed phosphate-buffered saline (PBS, 38 °C) containing 100 µg/mL penicillin, 100 µg/mL streptomycin and 100 µg/mL Amphotericin B (Biological industries, Kibbutz Beit-Haemek, Israel). Each adipose sample was cut into 5 pieces (each about 50 g) and placed in prewarmed PBS (38 °C) containing 100 µg/mL penicillin, 100 µg/mL streptomycin and 100 µg/mL Amphotericin B (Biological industries, Israel) and transported to the cell culture laboratory within one hour after sampling. The study was in accordance with the regulations of Ministry of Health, Israel. Certificate Nu: #80. Adipose tissue was collected after cows were slaughtered by a certified worker at Haifa commercial slaughterhouse (32694 IL).

### 2.2. Isolation of Mature Adipocyte and Induction of Dedifferentiated Adipocyte-Derived Progeny (DFAT) Cells

Subcutaneous adipose tissue samples were washed with PBS containing 100 µg/mL penicillin, 100 µg/mL streptomycin and 250 µL/mL Ampho-B and placed in culture hood. About 10 g of tissue was collected from the samples after trimming into a dish (100 mm). From each animal, five grams of tissue (about 3 mm) was transferred into fresh sterilized 50 mL centrifuge tube (*n* = 5). To this, prewarmed PBS medium was added, containing 2.5 µg/µL of collagenase enzyme type I, 3.75 g of BSA and 225.25 mg D-Glucose (Sigma Aldrich, Rehovot, Israel). The method for isolation of DFAT cells was adapted according to Wei, et al. [32] with some modifications. The tissue–collagenase mixture was incubated at 37 °C for one hour in a shaking water bath. After collagenase digestion, the contents of the tubes were filtered using 200 μM sterile metal mesh into fresh sterile 50 mL centrifuge tubes. The collagenase treated tissue in the tubes was separated into three layers by centrifugation at 400× *g* for 15 min. Three layers include adipocytes in the top layer, collagenase in the middle layer and the pellet. The top layer was transferred into five fresh sterile 50 mL centrifuge tubes each containing 20 mM HEPES buffer, 2.4 mM L-glutamine solution, 8 g/L D + Glucose, 5% Fetal calf serum, 1.6 g D-Glucose and 1% Penicillin Streptomycin-Amphotericin B Solution all in DMEM F-12 (Biological Industries, Kibbutz Beit-Haemek, Israel). A precoated Poly-lysine flask was completely filled with culture media, closed tightly and then inverted in cell culture incubator (37 °C, 5% CO_2_) until the cells became adherent and after reaching adhesion, the flasks were reverted and the cells were handled for further study.

### 2.3. Treatment of DFAT Cells with Resveratrol (RSV)

A preliminary calibration experiment was done by using RSV (98% RSV powder, Xi’an Green Bio-Tech Co., Ltd., Xi’an, Shaanxi, China) at two different concentrations: 100 and 200 µM to examine the minimal dose required for effects on cell viability and lipid droplets of DFAT cells, during 24 and 48 h. The RSV was reconstituted in 70% ethanol; therefore, the untreated cells were supplemented with the same amount of ethanol for equivalent conditions. At the end of the experiment, the medium was placed at −80 °C for further analysis.

### 2.4. Morphological Observation and Oil Red O (ORO) Staining of DFAT Cells

The DFAT cells were cultured in a 37 °C incubator with 95% air and 5% CO_2_, observed for morphological changes at regular intervals by using phase contrast microscope. After the treatment with RSV (100 and 200 µM) for 24 and 48 h, cells were stained with ORO (oil red-O, (Sigma Aldrich, Rehovot, Israel) for lipids as described by Kinkel, et al. [33]. The photos of the cells were taken with a Sony RGB color sensor (3/4-chip) connected to a phase contrast microscope (Nikon, Agentek, Yakum, Israel). DAPI staining method was used to stain the nuclei of the cells [34].

### 2.5. Antioxidant Activity of RSV

#### 2.5.1. Lipid Peroxidation Assay (TBARS)

Concentrations of malondialdehyde (MDA) are an index of lipid peroxidation and oxidative stress. In this thiobarbituric acid reactive substances (TBARS) assay, 20% trichloroacetic acid (TCA, Sigma Aldrich, Rehovot, Israel) in DDW was added in a ratio of 1:1 to the sample in a tube and MDA was used as a standard in serial dilutions. The samples were kept on ice for 15 min and later centrifuged at 10,000 rpm (4 °C) for 10 min. The supernatant collected into a fresh tube and 2-Thiobarbituric Acid (0.5% TBA in the TCA solution, (Sigma Aldrich, Rehovot, Israel) was added and boiled at 80 °C for 30 min and then cooled on ice for 15 min. Spectrophotometric measurements at 540 nm were performed to analyze the results. 

#### 2.5.2. Oxygen Radical Absorbance Capacity (ORAC)

Oxygen radical absorbance capacity (ORAC) was measured in according to Huang, et al. [35]. In brief, 25 µL of 73 mM AAPH (2,2′-Azobis(2-methylpropionamidine) dihydrochloride, (Sigma Aldrich, Rehovot, Israel) was added into each well in a 96-well microplate containing 25 µL of conditioned medium sample with the addition of 150 µL of 10 nM fluorescein. The intensity of the fluorescence samples was evaluated using Infinite M200 Pro (Tecan, Neotec, Kefar Sava, Israel).

### 2.6. Resveratrol Effects on the DFAT Cells under Different Thermal Treatments

The DFAT cells were placed in 6-well plates (3 × 10^5^ cells in each well) and incubated under experimental conditions including isothermal (ISO; 37 °C for 48 h), short heat stress (SH; 37 °C for 47 h and 41.2 °C for last 1 h) and long heat stress (LH; 37 °C for 32 h followed by last 16 h at 41.2 °C). Dairy cows’ body temperature is approximately 38 °C but because we used the adipose from the hind leg subcutaneous area, the local temperature is 37 °C according to our preliminary data. The cells were cultured at 41.2 °C to represent an SH for one hour, and LH for 16 h. Resveratrol (RSV; 100 µM) was added to the culture medium and cells were incubated for 48 h under ISO, SH and LH conditions. From the results of calibration process, the 100 µM concentration of RSV for 48 h was chosen as the desired experimental dosage for gene expression and protein abundancy during in vitro HS conditions. RSV treatment was estimated in three replicates for ISO, SH and LH thermal treatment, of the five different cows. According to our experiment six different treatments included a control group containing Isothermal DFAT cells with no RSV, Isothermal DFAT cells with RSV, SH DFAT cells without RSV, SH DFAT cells with RSV, LH DFAT cells without RSV and LH DFAT cells with RSV treatment. 

### 2.7. Extraction and Quantification of RNA by RT- PCR from Treated DFAT Cells

The total RNA was extracted from DFAT cells collected from each well; ISO, SH and LH from control and RSV treatment (100 µM) for 48 h, using Total RNA Purification Micro Kit (Qiagen, Hilden, Germany), following the manufacturer’s instructions. The integrity and concentration of the RNA was verified and quantified using a Nanodrop 1000 spectrophotometer (Thermo Fischer Scientific, San Jose, CA, USA). First-strand cDNA was generated by a cDNA reverse transcription kit and the process was undertaken according to the manufacturer’s instruction using the RevertAid cDNA Synthesis Kit (Thermo Fischer Scientific, San Jose, CA, USA). Quantitative detection of the gene expression was carried out using a real-time PCR (StepOnePlus, Applied Biosystems, San Jose, CA, USA) using the SYBR green PCR mix (Invitrogen, Carlsbad, CA, USA). The relative expressions of BCL2 Associated Agonist of cell death (*BAD*), BCL2 Associated x Protein (*BAX*), chemotactic C-C motif chemokine ligand 2 (*CCL2*), Fatty Acid Synthase (*FASN*), Forkhead Box O1 (*FOXO1*), Forkhead Box O3 (*FOXO3*), Heat Shock transcription Factor 1 (*HSF1*), Hormone Sensitive Lipase (*LIPE*), Interleukin 1 Beta (*IL1β*), Monoglyceride Lipase (*MGLL*), Proliferating Cell Nuclear Antigen (*PCNA*), Perilipin 1 (*PLIN*1), Peroxisome Proliferator Activated Receptor Gamma (*PPARG*), Sirtuin 1 (*SIRT1*), Superoxide Dismutase1 (*SOD1*) and Stress Induced Phosphoprotein 1 (*STIP1*) genes were quantified. *BRPS2* gene was chosen as a reference gene after examining several candidate genes in DFAT samples (*UXT*, *EIF4E*, *GAPDH* and β-actin). The list of primers is described in Table 1.

### 2.8. Immunoblot Analysis of FASN

The protein homogenization buffer was prepared (400 μL containing 10% SDS, 0.5 M EDTA, 1 M sodium fluoride, 1 M HEPES, 1 μL/mL phosphatase inhibitor and 1 μL/mL protease inhibitor (Sigma Aldrich, Rehovot, Israel) and added to the cells, stored at −80 °C for further protein extraction. The protein extraction protocol for the Western blotting was according to Daddam, et al. [36]. The DFAT cells treated with RSV (100 µM) and controls were collected from three replicates under SH and LH for 48 h and protein was isolated from each sample for Western blot analysis. Bradford assay was performed to assess the protein concentration in the samples. Following electrophoresis, protein blots were transferred to membrane and blocked with 1% BSA solution (Sigma Aldrich, Rehovot, Israel) for 1 h. Blots were incubated with specific primary antibodies (*FASN*-1:2000, ab99359, Abcam Biotech, Cambridge, UK; and β-Actin −1:1000, 4967, Cell Signalling Technology, Danvers, MA, USA) followed by incubation at 4 °C with goat anti-rabbit IgG horseradish peroxidase-linked secondary antibody (1:10,000, 111-035-003, Jackson Immunoresearch West Grove, PA, USA). Proteins were detected with the chemiluminescence substrate Detection Kit (Thermo Fischer Scientific, San Jose, CA, USA). Specific band signals were normalized to β-actin. Data was processed and analyzed by densitometry using ImageJ software (NIH, Bethesda, MD, USA).

### 2.9. In Silico Inhibitory Studies of RSV

The inhibitory studies of RSV were confirmed by docking to *FASN* structure using in silico methods. The structure of RSV was designed and optimized using chemsketch software and *FASN* structure was predicted using homology modeling. 

#### 2.9.1. Homology Modeling and Active Site Prediction of *FASN*

From the UNIPROT database, the amino acid sequence of *FASN* from *Bos taurus* (Uniprot_KB Accession Id: Q71SP7) was acquired as there was a lack of availability of *FASN* structure in the structural database. For domain identification, the *FASN* sequence in FASTA format was submitted to SBASE (pongor.itk.ppke.hu accessed on 5 January 2021) server, and to find out the related protein structure the predicted domains were searched by BLAST (blast.ncbi.nlm.nih.gov accessed on 5 January 2021) against PDB [37]. Using the default parameters in ClustalX, the sequence of template aligned with the target sequence [38]. Homology modeling was done by using MODELLER9V7 software to construct the initial model of *FASN*. This software generated 50 models for *FASN*, and the lowest energy model was selected for studies depending upon the lesser objective function. Later, by molecular dynamics simulation, the protein was stabilized by adding hydrogens to the three-dimensional structure. With the help of the NAMD 2.8 and CHARMM27 force field, MD simulations of the predicted model were performed [39]. In molecular dynamics studies, the structure of *FASN* with lesser root mean square deviation (RMSD) is achieved, and to examine the Stereochemical quality of protein structures; it is then determined by Ramachandran plot, using PROCHECK server. Later the environment profile was checked using structure evaluation server ERRAT [40]. Depending upon the template’s structural comparison, the possible binding sites of *FASN* were identified by using CASTp server (sts.bioe.uic.edu/castp accessed on 22 February 2021) [41].

#### 2.9.2. Docking Studies with Resveratrol

Using GOLD 3.0.1 software, the insight into the binding conformation of Resveratrol was gained by performing docking studies [42]. In this method, Resveratrol compound was docked to the active site of *FASN* to study the possibility of inhibitory activity of the RSV on the *FASN* activity. After docking, the individual binding poses of each protein–protein complex was selected and their binding energies were studied. The most energetic conformation of the complex was selected and docking studies were analyzed [43].

### 2.10. Statistical Analysis

The gene expression (∆∆CT; RQ) and protein abundance were examined using the GLM procedure of SAS (version 2.2, 2003).

The model used was:

Y_ijkl_ = µ + T_i_ + L_j_ + C(T × L)_ijk_ + E_ijkl_

where µ = overall mean; T_i_ = RSV effect, i = 1 to 2; L_j_ = heat treatment, j =1 to 3; C(T × L)_ijk_ = RSV*heat treatment; E_ijkl_ = random residual.

For gene expression analysis, due to variation in the RNA amount between samples, the RNA amount that was extracted from each sample was used as co-variance in the model. For each gene, the effect of RSV, heat treatments, and the interaction RSV × heat were considered for statistical analysis. Average values were corrected to the average RQ in the control treatment for each gene. The MDA and ORAC analysis was done using Student’s *t*-test in the excel program (2010). The data is shown as average values ± average standard error. *p* value ≤ 0.05 was labeled as statistically significant and <0.1 was labeled as statistical tendency.

## 3. Results

**Figure 1:**
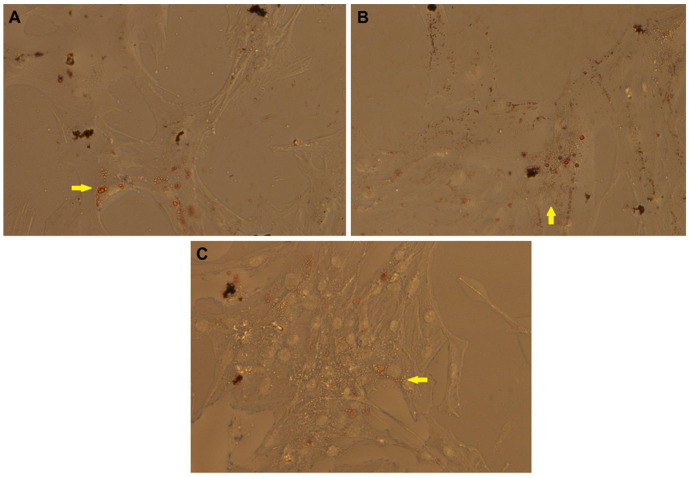
Representative Oil Red O staining of dedifferentiated adipocyte-derived progeny (DFAT) cells at 48 h. (**A**) DFAT cells untreated; (**B**) DFAT cells treated with 100 µM resveratrol; (**C**) DFAT cells treated with 200 µM resveratrol. Lipid droplets stained in yellow color (marked).

Bovine dedifferentiated adipocyte-derived progeny (DFAT) cells were generated from mature adipocytes of subcutaneous adipose tissue and treated with resveratrol (RSV) at concentrations of 100 and 200 µM for 48 h. According to cell counts (Supplementary Materials Figure S1) and 4′6-Diamidino-2-Phenylindole (DAPI) staining (Supplementary Materials Figure S2), DFAT cells were viable in control and RSV treatments (100 and 200 µM for 48 h). As shown in Figure 1, Oil Red O staining showed lipid droplets of DFAT cells from the control, 100 μM and 200 μM RSV treatments.

### 3.1. Antioxidant Activity of Resveratrol (RSV) in DFAT Cells

**Figure 2:**
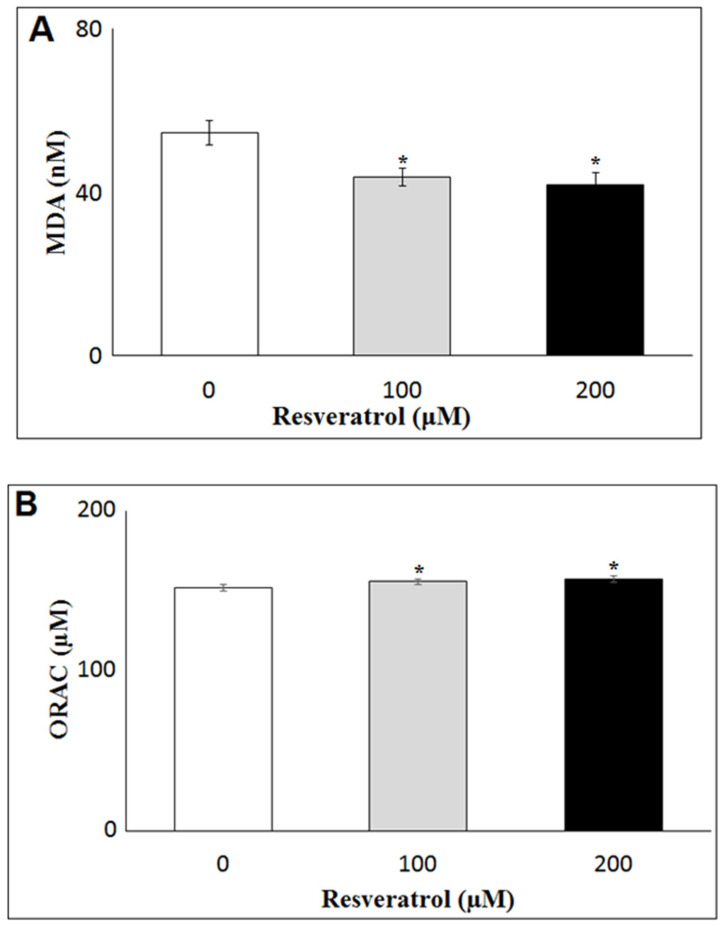
Indicators of oxidative stress in medium of DFAT cells treated with RSV at 100 and 200 µM by measuring (**A**) malondialdehyde (MDA) content and (**B**) oxygen radical absorbance capacity (ORAC) at 48 h. * indicates significant difference (*p* < 0.05).

The antioxidant effect of RSV on DFAT was studied by measuring the levels of MDA, an oxidative stress marker, and total antioxidant capacity by ORAC in the conditioned medium of DFAT cells. MDA levels were significantly decreased at the concentrations 100 µM (*p* = 0.01) and 200 µM (*p* = 0.05) RSV treatment compared to control (Supplementary Materials Table S1 and Figure 2A). ORAC levels were significantly increased at 100 µM (*p* = 0.008) and 200µM (*p* = 0.05) RSV treatments compared to the control (Supplementary Materials Table S1 and Figure 2B), thus indicating the antioxidative activity of RSV.

According to these findings, we chose the minimal effective concentration of 100 µM RSV to examine the effects of RSV, heat treatments (isothermal (ISO), short heat (SH) or long heat (LH) conditions), and their interaction on gene expression of markers related to oxidative stress, lipid metabolism, apoptosis, inflammation and SIRT1 signaling in DFAT cells (Supplementary Materials Table S2).

### 3.2. Effect of Resveratrol, Heat Treatments and Their Interaction on mRNA Expression Related to Oxidative Stress in Bovine DFAT Cells

**Figure 3:**
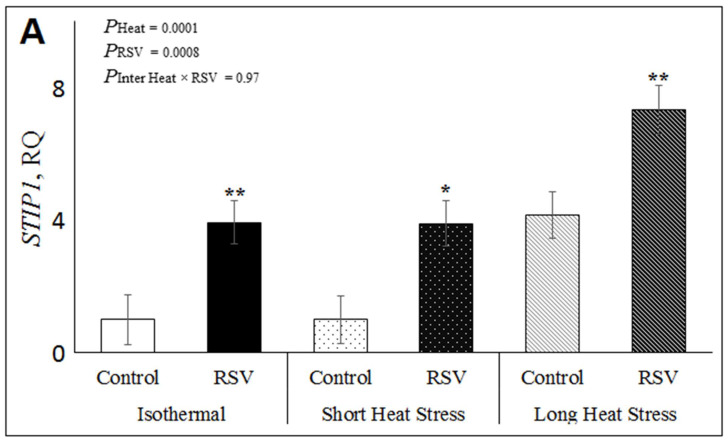

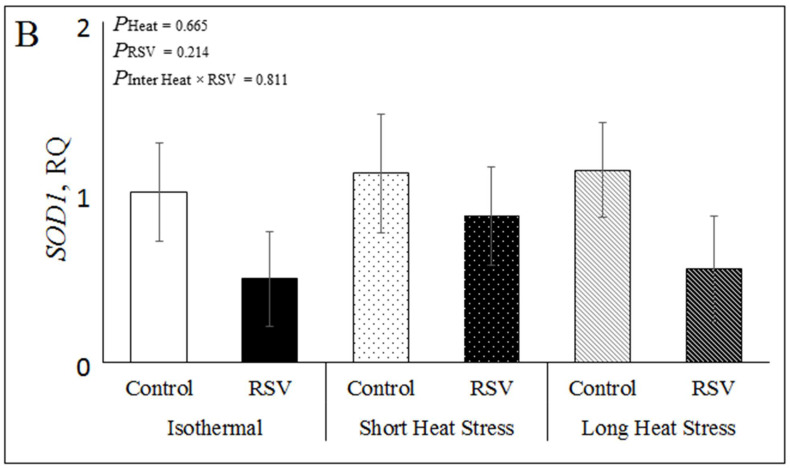
Effect of resveratrol (RSV; at 100 µM for 48 h) under isothermal (ISO; 37 °C for 48 h), short heat stress (SH; 37 °C for 47 h and 41.2 °C for last 1 h), or long heat stress (LH; 37 °C for 32 h followed by last 16 h at 41.2 °C) on relative expression of oxidative stress genes in DFAT cells. (**A**) Relative gene expression of stress induced phosphoprotein 1 (*STIP1*); (**B**) Relative gene expression of super oxide dismutase 1 (*SOD1*). *P*_heat_ is the significance of heat stress treatments, *P*_RSV_ is significance for RSV treatment and *P*_inter Heat × RSV_ is significance of interaction of Heat × RSV. Within each heat treatment, * indicates *p* < 0.05 and ** indicates *p* < 0.01 between control and RSV treatments.

To study the in vitro effects of RSV and heat stress on oxidative stress in the bovine DFAT cells, we examined the relative expressions of heat shock factor 1 (*HSF1)*, super oxide dismutase 1 (*SOD1*) and stress induced phosphoprotein 1 (*STIP1*) genes under ISO, SH and LH conditions (Supplementary Materials Table S2). As shown in Figure 3A, the relative mRNA expression of *STIP1* was increased by RSV (*P*_RSV_ = 0.0008), both in ISO (*p* = 0.009), SH (*p* = 0.011) and LH conditions (*p* = 0.006; Figure 3A). In addition, heat treatments significantly increased *STIP*1 expression (*P*_heat_ < 0.0001), and in LH (*p* < 0.0001) when compared to ISO, while the interaction RSV × Heat was not significant for *STIP1* (*p* = 0.97; Figure 3A). However, the relative expression of *SOD1* was not significantly affected by RSV, heat treatments or their interaction in DFAT cells (Figure 3B), nor was the relative expression of *HSF1* (Supplementary Materials Table S2).

### 3.3. Effect of Resveratrol, Heat Treatments and Their Interaction on mRNA Expression Related to Lipid Metabolism in Bovine DFAT Cells

**Figure 4:**
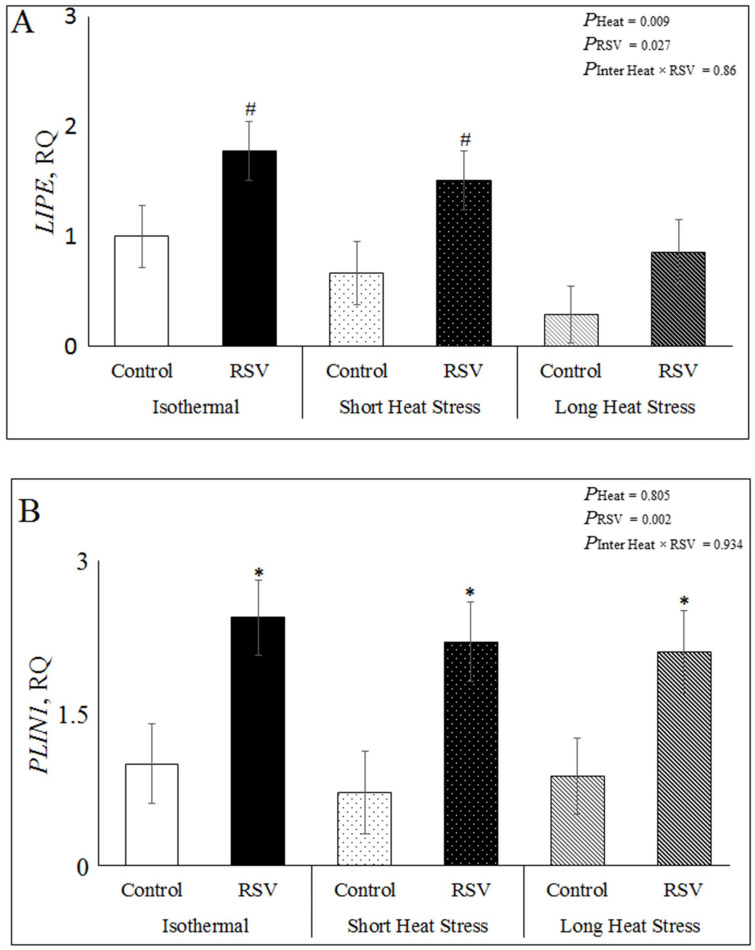

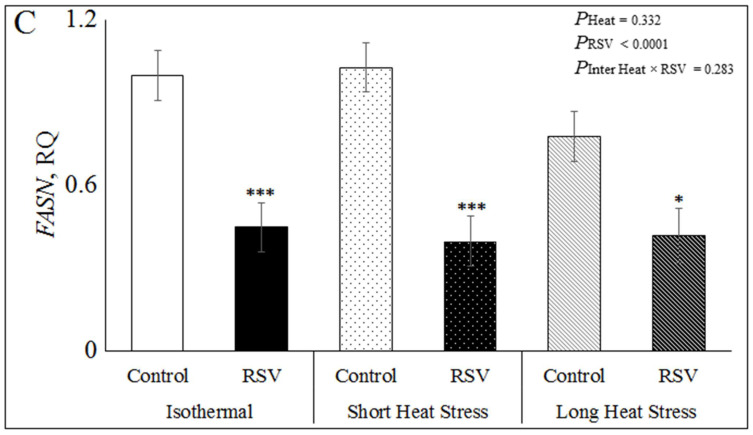
Effect of resveratrol (RSV; at 100 µM for 48 h) under isothermal (ISO; 37 °C for 48 h), short heat stress (SH; 37 °C for 47 h and 41.2 °C for last 1 h), or long heat stress (LH; 37 °C for 32 h followed by last 16 h at 41.2 °C) on relative expression of lipid metabolism in DFAT cells. (**A**) Relative gene expression of hormone sensitive lipase (*LIPE*); (**B**) Relative gene expression of perilipin 1 (*PLIN1*). (**C**) Relative gene expression of fatty acid synthase (*FASN*). *P*_heat_ is the significance of heat stress treatments, *P*_RSV_ is significance for RSV treatment and *P*_inter Heat × RSV_ is significance of interaction of Heat × RSV. Within each heat treatment, * indicates *p* < 0.05, *** indicates *p* < 0.001 and # indicates *p* = 0.06 between control and RSV treatments.

The in vitro effects of RSV and HS on lipid metabolism including lipolysis and lipogenesis in the bovine DFAT cells were examined by hormone sensitive lipase (*LIPE*), monoglyceride lipase (*MGLL*)*,* perilipin 1 (*PLIN1*) and fatty acid synthase (*FASN*) relative expressions under ISO, SH and LH conditions. Treating DFAT cells with RSV significantly increased the relative expression of the lipolytic enzyme *LIPE* (*P*_RSV_ = 0.027), and it tended to increase in ISO (*p* = 0.06) and SH (*p* = 0.06), but not in LH (*p* = 0.19; Supplementary Materials Table S2 and Figure 4A). However, heat treatments significantly decreased *LIPE* expression (*P*_heat_ = 0.009) in LH (*p* = 0.003) but not in SH when compared to ISO. However, the interaction RSV × Heat was not significant for *LIPE* (*p* = 0.86; Figure 4A; Supplementary Materials Table S2). Treating DFAT cells with RSV significantly increased the expression of *PLIN1 (P*_RSV_ = 0.002), in ISO (*p* = 0.01), SH (*p* = 0.01) and LH conditions (*p* = 0.03; Figure 4B and Supplementary Materials Table S2). Heat treatments (*P*_heat_ = 0.805) and the interaction RSV × Heat (*P*_inter_ = 0.934) had no significant effect on *PLIN1* expression (Figure 4B and Supplementary Materials Table S2). The relative gene expression of the lipogenic enzyme *FASN* significantly decreased by RSV treatment (*P*_RSV_ < 0.0001), both in ISO (*p* = 0.0004), SH (*p* = 0.0001) and LH (*p* = 0.023; Figure 4C and Supplementary Materials Table S2). Heat treatments (*P*_heat_ = 0.332) and the interaction RSV × Heat (*P*_inter_ = 0.284) had no significant effect on *FASN* expression (Figure 4C and Supplementary Materials Table S2). The relative expression of the enzyme *MGLL* was not significantly affected by RSV, heat treatments or the interaction RSV × Heat in DFAT cells (Supplementary Materials Table S2).

### 3.4. Effect of Resveratrol, Heat Treatments and Their Interaction on mRNA Expression Related to Apoptosis, Inflammation and SIRT1 Signaling in Bovine DFAT Cells#

**Figure 5:**
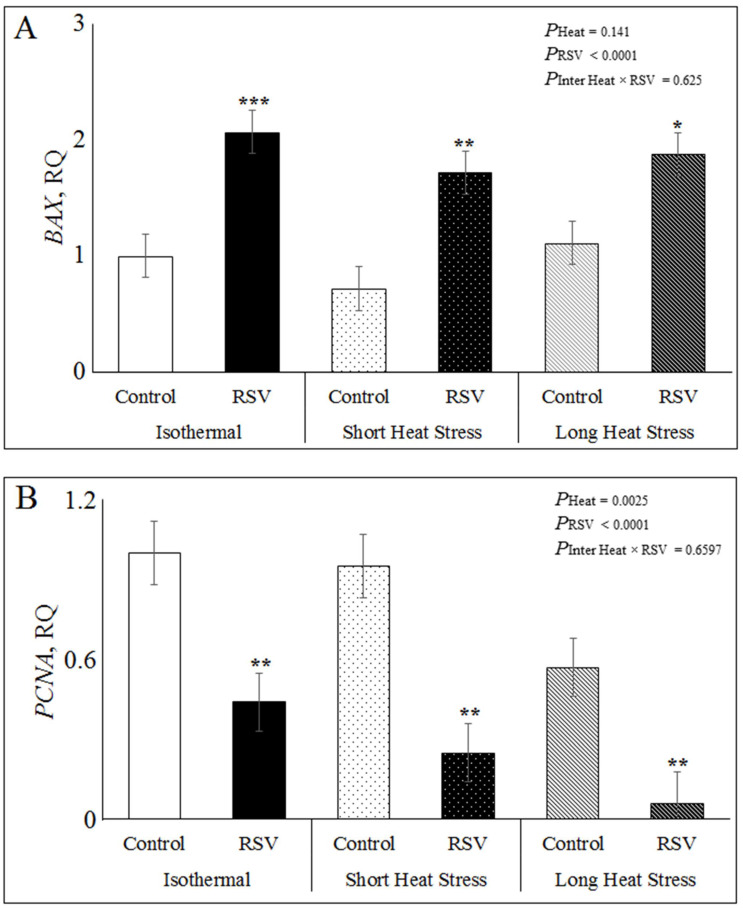

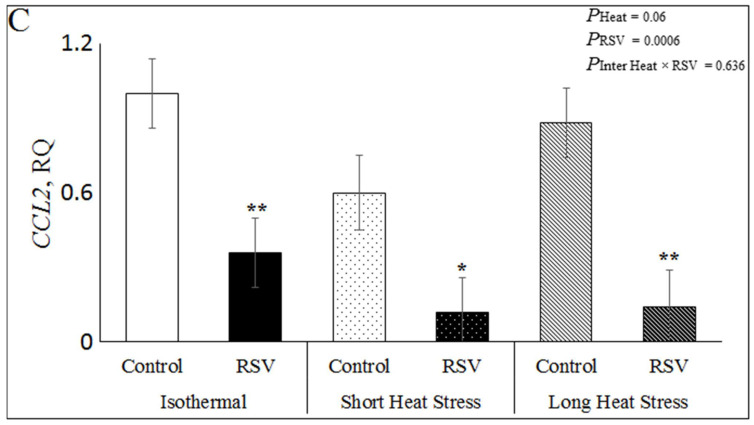
Effect of resveratrol (RSV; at 100 µM for 48 h) under isothermal (ISO; 37 °C for 48 h), short heat stress (SH; 37 °C for 47 h and 41.2 °C for last 1 h), or long heat stress (LH; 37 °C for 32 h followed by last 16 h at 41.2 °C) on relative expression of lipid metabolism in DFAT cells. (**A**) Relative gene expression of stress induced BCL2-associated-x-protein (*BAX*). (**B**) Relative gene expression of proliferating cell nuclear antigen (*PCNA*). (**C**) Relative gene expression of chemokine-ligand 2 (*CCL2*)*. P*_heat_ is the significance of heat stress treatments, *P*_RSV_ is significance for RSV treatment and *P*_inter Heat × RSV_ is significance of interaction of Heat × RSV. Within each heat treatment, * indicates *p* < 0.05, ** indicates *p* < 0.01, and *** indicates *p* < 0.001 between control and RSV treatments.

Treating DFAT cells in vitro with RSV treatment, HS and these interactions on apoptosis in the bovine DFAT cells was studied by the relative expressions of BCL2-associated-agonist-of-cell-death (*BAD*), BCL2-associated-x-protein (*BAX*) and proliferating-cell-nuclear-antigen (*PCNA*) genes under ISO, SH and LH conditions. As shown in Figure 5A, in all heat treatments, RSV significantly increased *BAX* expression (*P*_RSV_ < 0.0001), in ISO (*p* = 0.0004), SH (*p* = 0.001) and LH conditions (*p* = 0.01; Figure 5A). However, the heat treatments (*P*_heat_ = 0.141) and the interaction RSV × Heat (*P*_inter_ = 0.625) had no significant effect on *BAX* gene expression (Figure 5A). In addition, RSV significantly decreased *PCNA* expression (*P*_RSV_ < 0.0001) in ISO (*p* = 0.003), SH (*p* = 0.0007) and LH (*p* = 0.007; Figure 5B and Supplementary Materials Table S2). Moreover, heat treatments had a significant effect on *PCNA* expression (*P*_heat_ = 0.0025), where the *PCNA* decreased in SH (*p* =0.28) and LH (*p* = 0.0007) when compared to ISO. The interaction RSV × Heat was not significant for *PCNA* expression (*p* = 0.65; Figure 5B). The relative expression of *BAD* was not affected by RSV, heat treatments or their interaction (Supplementary Materials Table S2).

The gene expression of the pro-inflammatory gene chemotactic C-C motif chemokine ligand 2 *(CCL2*) significantly decreased by RSV treatment (*P*_RSV_ < 0.0006), in ISO (*p* = 0.006), SH (*p* = 0.04) and LH (*p* = 0.002; Supplementary Materials Table S2 and Figure 5C). Additionally, heat treatments (*P*_heat_ = 0.06) tended to decrease *CCL2* expression in SH (*p* = 0.2), while LH (*p* = 0.22) increased *CCL2* when compared to ISO, however, the interaction RSV × Heat was not significant (*P*_inter_ = 0.636; Figure 5C). The relative expression of interleukin-1β (*IL1-β)* was not significantly affected by RSV, heat treatments and interaction in DFAT cells (Supplementary Materials Table S2).

Among SIRT1 signaling genes, sirtuin 1 (*SIRT1*), forkhead box O1 (*FOXO1*), forkhead box O3, (*FOXO3*) and peroxisome proliferator activated receptor Gamma (*PPARγ*), only *FOXO3* tended to be lower in RSV vs. control, with no effect of heat treatments or their interaction (Supplementary Materials Table S2).

### 3.5. Protein Abundance of FASN in DFAT Cells Treated with RSV under In Vitro HS Conditions

**Figure 6:**
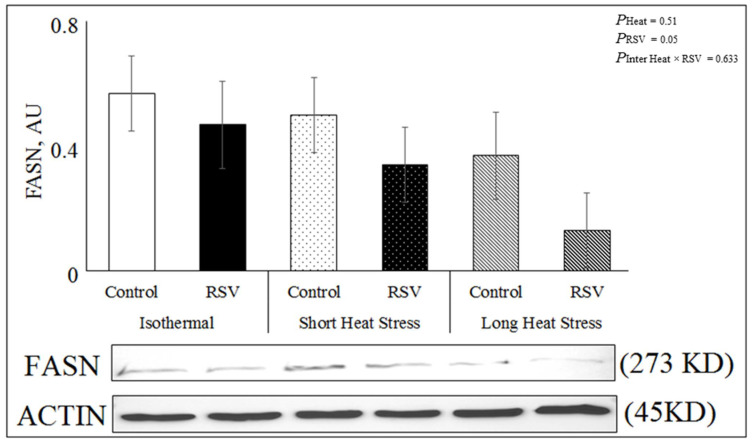
Effect of RSV (100 µM for 48 h) on the abundance of FASN under isothermal (ISO; 37 °C for 48 h), short heat stress (SH; 37 °C for 47 h and 41.2 °C for last 1 h), or long heat stress (LH; 37 °C for 32 h followed by last 16 h at 41.2 °C). Actin used as reference band.

Treating DFAT cells with RSV significantly decreased the abundance of *FASN* (*P*_RSV_ = 0.05) across heat treatments (Figure 6). However, both short and long heat treatments (*P*_heat_ = 0.51) had no effect on the abundance of *FASN*, and the interaction RSV × Heat was not significant (*P*_inter_ = 0.63).

### 3.6. Inhibitory Activity of Resveratrol on FASN by Docking Studies

**Figure 7:**
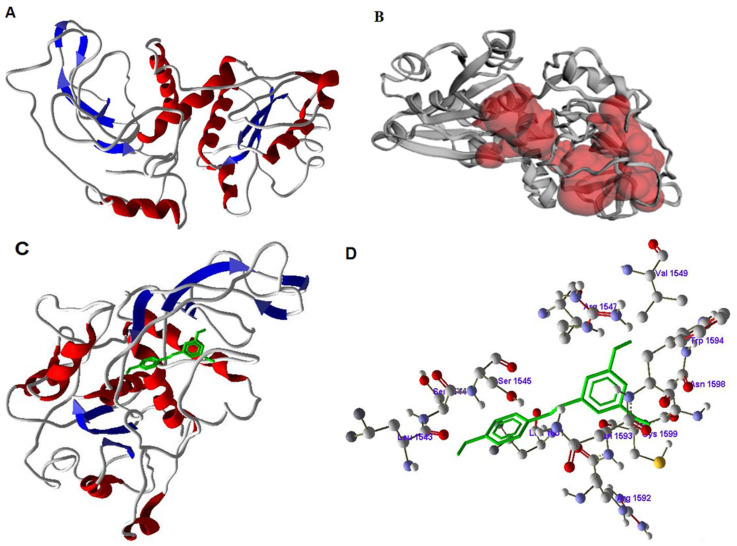
Inhibitory studies of resveratrol with fatty acid synthase (*FASN*). (**A**) Structure of *FASN* with helices (red color) and sheets (blue color), (**B**) active site of *FASN* in red color pocket in the structure, (**C**) docking of Resveratrol (green color) with FASN, and (**D**) amino acids of *FASN* involved in docking with resveratrol.

In order to find the possible inhibitory activity of RSV on *FASN*, a three-dimensional structure was modeled using *FASN* protein sequence from *Bos taurus*. *FASN* contains Beta-ketoacyl synthase domain (1–239; 243–405 amino acids), Acyl transferase region (492–808 AA), Transporter-associated region (877–943 AA), Pyridine nucleotide-disulphide oxidoreductase, NAD-binding region (1081–1123 AA), Methyltransferase type 12 (1247–1337 AA), Bacterial type II secretion system protein (1514–1575 AA), Alcohol dehydrogenase, zinc-binding domain (1669–1818 AA), Short-chain dehydrogenase/reductase (1881–2049 AA), Phosphopantetheine-binding domain (2127–2193 AA) and Thioesterase domain (2239–2485 AA). The model was generated by using a template structure of Mammalian Fatty Acid Synthase (PDB code: 2VZ8_A) of *Sus scrofa* collected from protein data bank as a result of BLAST search where the template showed maximum similarity (80.4%) with *FASN* from *Bos taurus*. The final *FASN* structure was obtained by molecular dynamics and validated using Ramachandran plot server using PROCHECK program (Figure 7A).

The binding sites of *FASN* were searched using CASTp server as well as comparing with template (Figure 7B). The Resveratrol was designed and optimized by Chemsketch software and docked to *FASN* binding region using GOLD 3.0.1 software. The selected docked conformations of RSV with the *FASN* binding site are depicted in Figure 7C,D. The amino acids SER1545, ARG1547, VAL1549, TRP1594, ASN1598, ARG1592 and TYR1593 were involved in forming a strong hydrogen bond with RSV. Based on this, it is suggested that the inhibition of *FASN* was due to binding of RSV at active site and forming a complex with the active residues of *FASN*.

## 4. Discussion

In the present study, we examined the effects of the natural antioxidant RSV on bovine DFAT cells that were under isothermal, short or long heat stress in vitro. The antioxidant RSV is reported to possess many health benefits and therefore it has been intensively investigated in human and rodent research [44,45]. Since HS causes reduction of antioxidant mechanism [46,47] we hypothesized that RSV may have a positive effect on DFAT cells under in vitro HS conditions. Indeed, in the current studies we found that supplementation of bovine DFAT cells with RSV reduced oxidative stress in isothermal conditions and increased the relative gene expression of *STIP1* also during HS. In addition, we found that RSV increased the expression of *LIPE* and *PLIN1* while reducing *FASN*, which may indicate of higher lipolysis to lipogenesis ratio in DFAT cells, also under HS conditions. Interestingly, possible docking of RSV to *FASN* may be related to the suppressive effect of RSV on *FASN* in these cells. The lower expression of *CCL2* may indicate an anti-inflammatory effect of RSV on DFAT cells. Another aspect of the present study is that we developed an efficient method to acquire cultures of DFAT cells from adipose tissue of Holstein cows, and this method can be used to examine the effects of other nutraceuticals on bovine DFAT cells.

The antioxidative properties of RSV have been previously demonstrated; RSV scavenges ROS and increases antioxidant levels in the organism during oxidative stress [48,49]. Superoxide (O_2_^−^) and hydrogen peroxide (H_2_O_2_) are oxygen radicals required in low levels for cell signaling processes [50], apoptosis, cell proliferation, induction of gene expression. Environmental factors like HS cause increase ROS levels and this imbalance of production causes negative effects in cells and alters normal cell function [51]. In this sense, ROS causes lipid peroxidation in the cells, which increases MDA formation [52]. Therefore, we investigated the oxidative status in the DFAT cells upon RSV treatment by measuring the MDA and ORAC levels. Our results show that the ability of the antioxidant defenses to neutralize ROS increased upon RSV treatment in DFAT cells, by decreasing MDA levels and increasing ORAC compared to control. Due to this it is suggested that RSV has capability to reduce oxidative stress in DFAT cells, although ROS levels were not directly measured. Based on the results of this study, RSV treatment improved antioxidant status of DFAT cells suggesting that the resveratrol-induced mechanism was independent of HS and similar to data obtained with other antioxidants [53].

HS has a direct effect on oxidative status and lipid metabolism of Holstein cows [29]. In adipose tissue of dairy cows, many proteins expressed are involved in metabolic reactions to stress [29]. Several points need to be taken into consideration in interpretation of results obtained from RSV treatment of DFAT cells. The HS used in this study may be different from the in vivo HS conditions. In order to understand the effect of HS on DFAT cells, we examined in vitro the effect of isothermal (ISO-37 °C), short heat stress (SH-41.2 °C for 1 h) and long heat stress (LH-41.2 °C for 16 h) conditions because in previous studies this temperature was chosen to represent extreme HS in bovine cells [29]. In the current study, adipocytes experienced acute HS, while the process likely occurring in vivo is different. Thus far, in the current experiments, cells change profoundly with long-term exposure to HS, but it is not yet known how they may become acclimated to prolonged HS exposure. We used subcutaneous adipose tissue to develop DFAT cells and the RSV effect in the study. However, it is unknown if other adipose depot-meditated cells (such as abdominal adipose) would react similarly to the HS. In the present study, exposure of DFAT cells to HS decreased the relative expression of *LIPE* and *MGLL* lipases, which may be related to a lower lipolytic response in adipose. In addition, HS increased the expression of *STIP1* but decreased the expression of *PCNA* that could suggest higher antioxidant coupled with lower cell proliferation in heat stressed DFAT cells. Thus, the current experiments provide novel and important data documenting the direct response of bovine DFAT cells to SH and LH conditions. Future experiments are now warranted to refine our understanding of these events in additional adipose depots to HS.

In the present study, RSV treatment increased *STIP1* expression in HS conditions, but SH decreased *STIP1* expression, whereas its expression was increased in LH. This differential pattern could be related to the time course of response to HS, where an acute HS induces a protective increase in *STIP1*, while following a longer heat exposure there is a downregulation of *STIP1* in DFAT cells. Among oxidative stress markers, stress-inducible protein 1 (*STIP1*) plays an important role in stress conditions. *STIP1* is a co-chaperone and acts as an adaptor that regulates Hsp90 to Hsp70–protein complexes in the cytoplasm [54]. It directs the functional cooperation of Hsp90 and Hsp70 during folding of various kinases and transcription factors including oncogenic proteins [55]. *STIP1* has been detected in all major tissues, such as the heart, liver, spleen, lung, kidney, brain, skeletal muscle and some mouse cell lines [55]. However, relatively little is known about *STIP1* in the adipocytes of dairy cows. Previous studies showed its expression in adipose tissue of dairy cows [3], but to the best our knowledge, this is the first time that the expression of *STIP1* in DFAT cells under different HS conditions was studied. We suggest that the changes in expression of *STIP1* indicate that RSV treatment may have decreased oxidative stress through *STIP1* mediated heat shock proteins. Another oxidative stress protein, superoxide dismutase 1 (*SOD1*), is known to have a capacity to limit the detrimental effects of ROS. The function of *SOD1* is to protect the cell when it undergoes oxidative stress [56]. However, in our study *SOD1* expression was not increased by RSV treatment or by HS in DFAT cells. This may suggest that RSV and HS possibly affect SOD1 at protein or activity level, and not at the mRNA level in this setting.

To investigate the effects of RSV treatment during HS on lipid metabolism of DFAT cells, we examined the expression of genes involved in regulation of lipolysis and lipogenesis. Our findings indicate that RSV treatment increased relative gene expression of *PLIN1*, *LIPE* and decreased *FASN*, indicating the possibility of enhanced lipolysis and reduced lipogenesis in bovine DFAT cells also under short- and long-term heat treatments. We found that RSV treatment significantly increased *LIPE* expression in DFAT cells under ISO and SH conditions, but no significant change was observed at LH. The role of *LIPE* in lipolysis is well known and its function in adipose tissue is controlled by translocation to the lipid droplet [57]; therefore, this finding indicates that RSV had a lipolytic effect of bovine DFAT cells. It may be postulated that LH has prevented the increase in *LIPE* expression, possibly as a means to limit lipolysis under chronic HS conditions, as was previously suggested [29]. In our study, RSV increased *PLIN1* expression in DFAT cells under isothermal, short and long HS conditions. The role of the *PLIN1* is to protect the lipid droplets from lipases, and during lipolysis phosphorylation leads to inactivation of *PLIN1* [58,59], which allows lipases to breakdown the lipid droplets by *LIPE* [60]. We suggest that the increase in *PLIN1* following RSV treatment may be a mechanism to limit lipolysis in adipocytes, however this issue warrants further investigation. We also found that the RSV treatment significantly decreased gene expression of *FASN* at different HS conditions. *FASN* synthesizes fatty acids and increases lipogenesis in adipocytes [61]. Our finding of lower *FASN* at gene and protein levels in RSV treated DFAT cells confirm the effect of RSV in inhibition of lipogenesis as reported in the literature [62]. Taken together, we observed that RSV increased lipolytic gene expression (*LIPE*) and decreased expression lipogenic gene expression and protein abundance (*FASN*) in bovine adipocytes and heat treatments or interaction had no significant effect on these patterns, indicating that RSV was effective in vitro also under HS conditions.

When examining the effects of RSV on cell apoptosis and proliferation, we found that the expression of *BAX* was higher and *PCNA* was lower in RSV treated DFAT cells. In apoptosis, p53 signals activate caspases through the interaction with Bax and Bcl-xL proteins [63]. *BAX,* the first member of Bcl-2 family is induced by p53 and accelerate apoptosis together with *BAD* [64]. Proliferating cell nuclear antigen (*PCNA*), a DNA polymerase delta variant, plays important roles in DNA replication and DNA repair controlled by p53 signaling mechanism [65]. Our findings may suggest that RSV treatment induced apoptosis and reduced proliferation in DFAT cells, by increasing *BAX* expression and reducing *PCNA* expression also under HS conditions. In addition, the current study showed that, regardless of HS treatments, RSV decreased in the expression of *CCL2* in DFAT cells. *CCL2* is a pro-inflammatory cytokine which can cause an accumulation of phagocytes in an infected area [66]. These findings reinforce that RSV is an anti-inflammatory agent in adipocytes under different heat conditions. Another study that examined the effects of RSV on cytokine levels in adipose tissue has shown that the RSV assists in lowering the expression of *IL1-β* levels in the visceral adipose tissue and not in the subcutaneous adipose tissue [67]. In the current research we collected the DFAT cells from subcutaneous adipose tissue and this can possibly explain the nonsignificant changes in the expression of *IL1-β* following RSV treatment.

In our study, no significant change was observed in *SIRT1* gene expression and related genes of *SIRT1* signaling where only *FOXO3* tended to be lower under RSV treatment at all HS conditions. In other studies examining adipocytes of dairy cows, the expression of *SIRT1* increased significantly in response to RSV treatment at different concentrations [68]. Our finding is inconsistent with other studies where RSV affected *SIRT1* gene expression even at low concentrations [69]. One of the possible reasons for the lack of RSV effect on *SIRT1* at the mRNA level may be due to components of the medium used to grow DFAT cells, and the genes examined to be involved in the *SIRT1* expression pathway. *SIRT1* binds to the genes in the pathway of fat accumulation of *PPARγ* and inhibits the expression. In the present study we did not find an effect of RSV on the expression of *PPARγ*. Forkhead Box O (FOXO) genes activate or inhibit other gene expressions in the nucleus and control apoptosis process. Among these, *FOXO1* and *FOXO3* expressions are in control of *SIRT1* expression and under stress conditions, *SIRT1* inhibits *FOXO1* and *FOXO3* gene expressions. Others demonstrated that RSV induced apoptosis in adipocytes from dairy cows, and it is known that *SIRT1* deacetylates *FOXO1* and induces apoptosis [70]. It was further shown that RSV treatment decreased mRNA levels of *FOXO1* following the activation of *SIRT1* [71]. In the present study, no effect of RSV was found on *FOXO1* expression, but it was found that RSV tended to reduce the expression of *FOXO3* in DFAT cells. Therefore, we suggest that the effects of RSV on DFAT cells may be directly by affecting *FASN* and other lipid related enzymes within the adipocyte (*PLIN1*, *LIPE*) and not via *SIRT1*, or alternatively that the effects on *SIRT1* are at the level of protein activity and not at the level of gene expression.

The lipogenic enzyme *FASN* is one of the rate-limiting enzymes in the de novo lipogenesis pathway in adipocytes of cattle [36]. Our data demonstrate that RSV significantly reduced the abundance of *FASN* under HS, which may indicate its role in reducing lipogenesis of bovine DFAT cells. Exposure of DFAT cells to high temperature causes a number of abnormalities in cell function, which include inhibition of protein synthesis, damage of protein structure and function leads to morphological changes caused in the structure of the intracellular skeleton. Previous studies showed RSV inhibitory activity by docking to different targets [72], and the activity of RSV as an antioxidant was studied previously through docking with specific target proteins [73]. In the present study we performed molecular docking of RSV on *FASN* related to lipogenesis. To study the interaction between two molecules, molecular docking is the widely used method in computational studies to develop structure-based drugs [74]. Additionally, in previous studies, RSV inhibitory and binding studies were documented [75,76]; however, the mechanism of *FASN* inhibition by RSV was not yet studied using docking methods. Using docking, we revealed that RSV binds to amino acids in active site of *FASN* and hence may inhibit *FASN* activity which may reduce lipogenesis. In most of the docking algorithms rigid or flexible protein structure is considered for the interaction with compounds. Generally, in flexible docking algorithm side chain fluctuations are taken into consideration and multiple confirmations of compounds are used to find a better docking complex. For this, a better structure of protein is needed to get good docking results and depends on quality of the protein structure. A highly reliable model is needed to generate an accurate hypothetical docking complex and here, as no PDB structure is available for *FASN*, we modeled a reliable structure and docked with RSV. The docking score of RSV and the binding poses suggest that RSV inhibits *FASN* by binding to the amino acids involved in the active site. Even though no experimental data is available regarding structures, the use of molecular docking can simulate interactions and binding scores. In order to improve docking studies towards accurate results, molecular dynamics of docked complex was used, and this corrects the inaccurate structural conformational change. The *FASN*-RSV complex formed after docking was improved by molecular dynamics simulations by correcting the interaction of the complex. From the molecular interactions it was confirmed that RSV formed a good ligand–protein complex with *FASN* showing the possible direct involvement in the mechanisms. This finding may indicate that RSV directly inhibits *FASN* in bovine DFAT cells.

## 5. Conclusions

This in vitro study demonstrates that the antioxidant RSV affects gene expression in bovine DFAT cells, by increasing the gene expression of the *LIPE* and decreasing the expression and abundance of *FASN*, which possibly enhances lipolysis and decreases lipogenesis under both isothermal and HS conditions. Moreover, docking studies showed that RSV docked to the active residues of *FASN*, confirming the interaction and its role in inhibition of *FASN*. Moreover, we found lower gene expression of pro-inflammatory *CCL2* in RSV treated cells, which may indicate an anti-inflammatory effect of RSV in bovine adipose tissue. Based on these findings, we suggest that RSV supplementation should be examined in vivo as a nutrigenomic dietary supplement that can reduce oxidative stress and increase lipid turnover in adipose tissue of heat stressed dairy cows.

## Figures and Tables

**Table 1:** List of primers used for mRNA expression in the DFAT samples.

Gene	GenBank Accession No.	Sequence 5′ > 3′^2^
*BAD*	NM_001035459.2	F: GAGGATGAGCGACGAGTTTC
		R: TCAACCAGGACTGGAGGAAG
*BAX*	NM_173894.1	F: AACATGGAGCTGCAGAGGAT
		R: CAGTTGAAGTTGCCGTCAGA
*BRPS2*	NM_001033613.2	F: GGAGCATCCCTGAAGGATGA
		R: TCCCCGATAGCAACAAACG
*CCL2*	NM_174006.2	F: ATCTCCATGCAGAGGCTGAT
		R: GCTTGGGGTCTGCACATAAC
*FASN*	NM_001012669.1	F: ACCTCGTGAAGGCTGTGACTCA
		R: TGAGTCGAGGCCAAGGTCTGAA
*FOXO1*	XM_025000053.1	F: TCACGCTGTCGCAGATTTAC
		R: TGCAGGGACAGATTATGACT
*FOXO3*	NM_001206083.1	F: CAGACAAACGGCTCACTCTG
		R: GGTTGTGCCGGATAGAGTTC
*HSF1*	NM_001076809.1	F: CCAGCAACAGAAAGTCGTCA
		R: GCATCAGGGGGATCTTTCTC
*LIPE*	NM_001080220.1	F: GAGTTTGAGCGGATCATTCA
		R: TGAGGCCATGTTTGCTAGAG
*IL1β*	NM_174093.1	F: TCCACCTCCTCTCACAGGAAA
		R: TACCCAAGGCCACAGGAATCT
*MGLL*	NM_001206681.1	F: GCAACCAGCTGCTCAACAC
		R: AGCGTCTTGTCCTGGCTCTT
*PCNA*	NM_001034494.1	F: AGGAGGAAGCTGTTGCCATA
		R: GGAGACAGTGGAGTGGCTTT
*PLIN1*	NM_001083699.1	F: AGACACTGCCGAGTATGCTG
		R: TGGAGGGAGGAGGAACTCTA
*PPARG*	NM_181024.2	F: TGCTGTGGGGATGTCTCATA
		R: GGTCAGCAGACTCTGGGTTC
*SIRT1*	NM_001192980.3	F: TGGCCAGCTAGACTTGCAAA
		R: AACTTGGACTCTGGCACGTT
*SOD1*	NM_174615.2	F: CGAGGCAAAGGGAGATACAG
		R: TCTCCAAACTGATGGACGTG
*STIP1*	NM_001035492.2	F: CTGGGGAATGAAGCCTACAA
		R: GGCTGCTTGGTTGGTTATGT

*BAD* = BCL2 Associated Agonist of cell death; *BAX* = BCL2 Associated x Protein; *BRPS2* = Bovine Ribosomal Protein S2; *CCL2* = Chemotactic C-C motif chemokine ligand 2; *FASN* = Fatty Acid Synthase; *FOXO1* = Forkhead Box O1; *FOXO3A* = Forkhead Box O3; *HSF1* = Heat Shock Protein Family A1 (70 KDa); *IL1β* = Interleukin 1 Beta; *MGLL* = Monoglyceride Lipase; *PCNA* = Proliferating Cell Nuclear Antigen; *PLIN1* = Perilipin1; *PPARG* = Peroxisome Proliferator Activated Receptor Gamma; *SIRT1* = Sirtuin 1; *SOD1* = Superoxide Dismutase1; *STIP1* = Stress Induced Phosphoprotein1.

## Data Availability

The data presented in this study are available in Supplementary Material.

## Supplementary Materials

The following are available online at https://www.mdpi.com/article/10.3390/antiox10060905/s1, Figure S1: Treatment of RSV at different concentrations of pre-adipose tissue, Figure S2: Cell viability of RSV treatment at different concentrations by DAPI staining, Table S1: RSV effect on the MDA and ORAC levels in DFAT cells, Table S2: RSV effect on the relative quantification (RQ) of different genes’ expression under short and long heat stress.
