# Supplementary material for: Antioxidant Resveratrol Increases Lipolytic and Reduces Lipogenic Gene Expression under In Vitro Heat Stress Conditions in Dedifferentiated Adipocyte-Derived Progeny Cells from Dairy Cows
Source: Antioxidants (Basel). 2021 Jun 3;10(6):905. doi: 10.3390/antiox10060905 (PMC8230285; doi:10.3390/antiox10060905)
Supplement: Supplementary file 1 [file antioxidants-10-00905-s001.zip › antioxidants-1218646-supplementary.pdf]

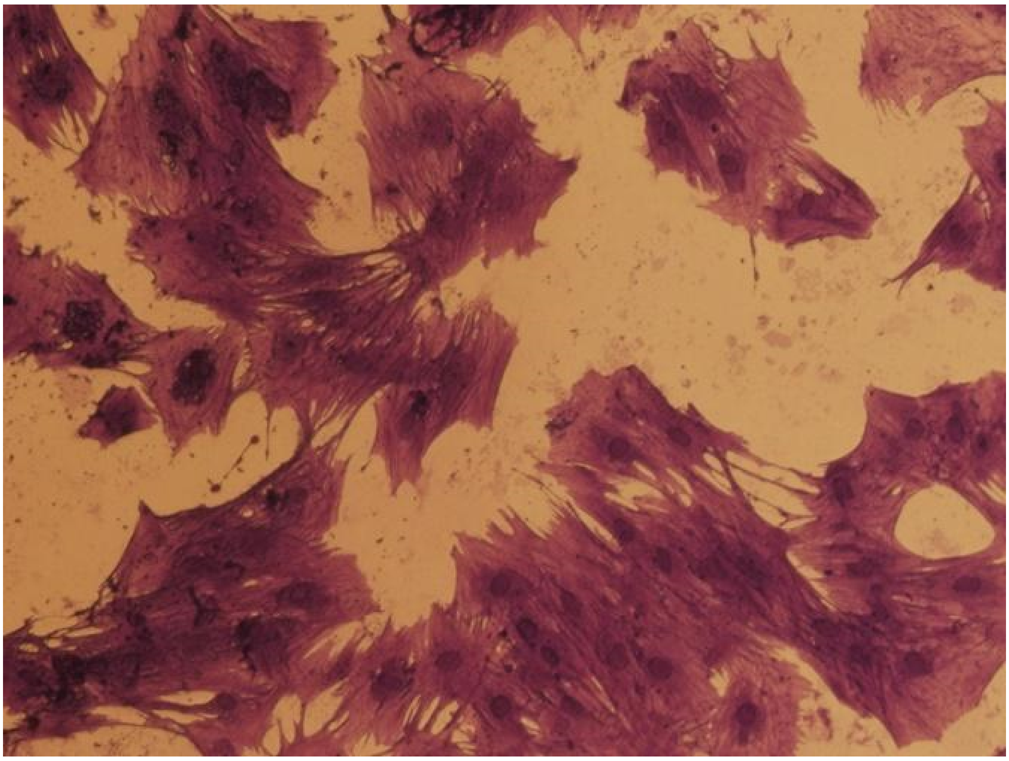

a) Control DFAT cells for at 24 hours

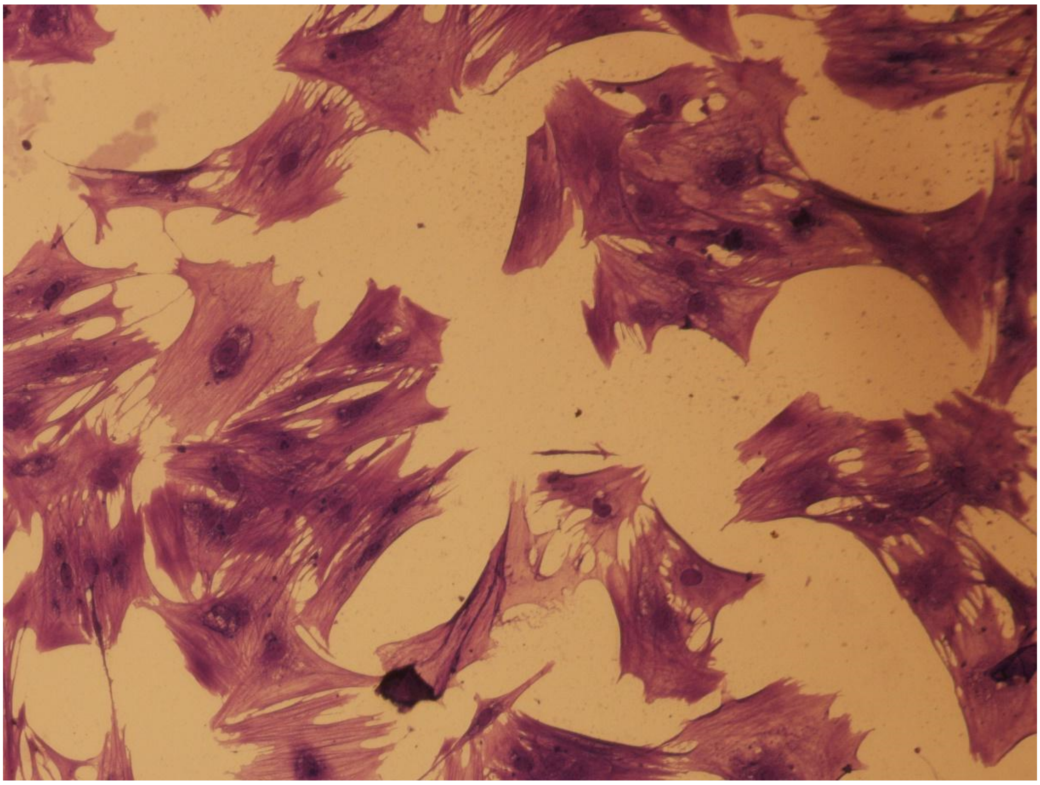

b) 100µM resveratrol treatment of DFAT cells at 24 hours

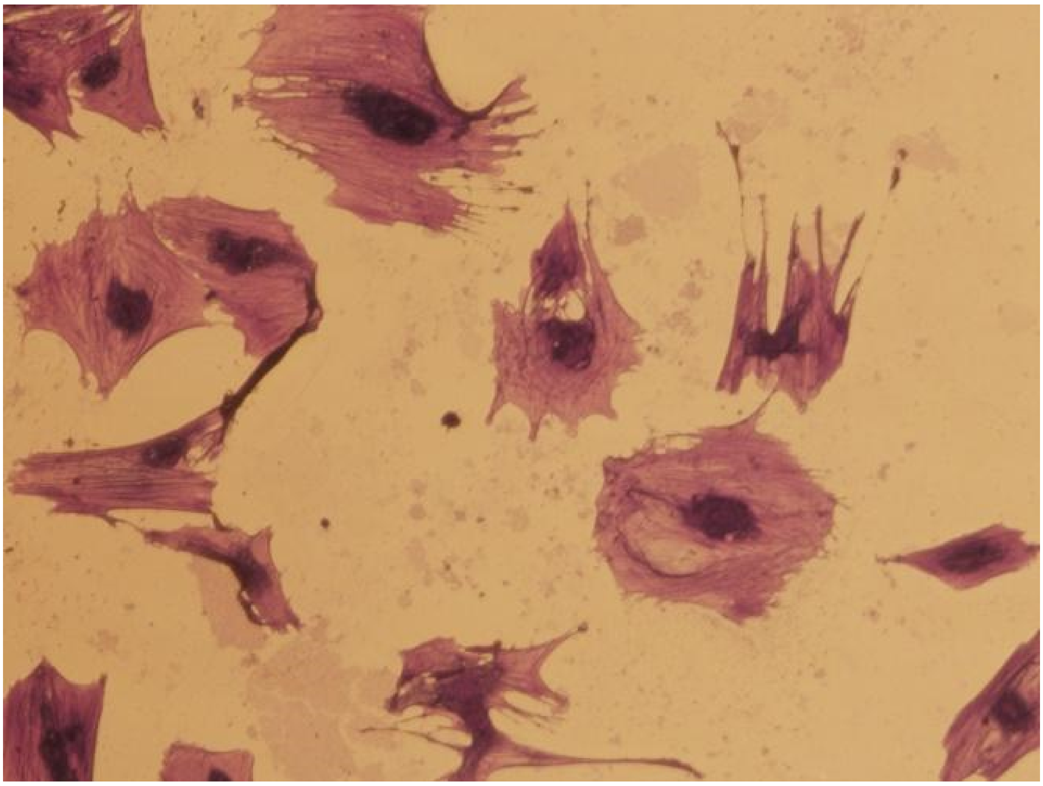

c) 200µM resveratrol treatment of DFAT cells at 24hours

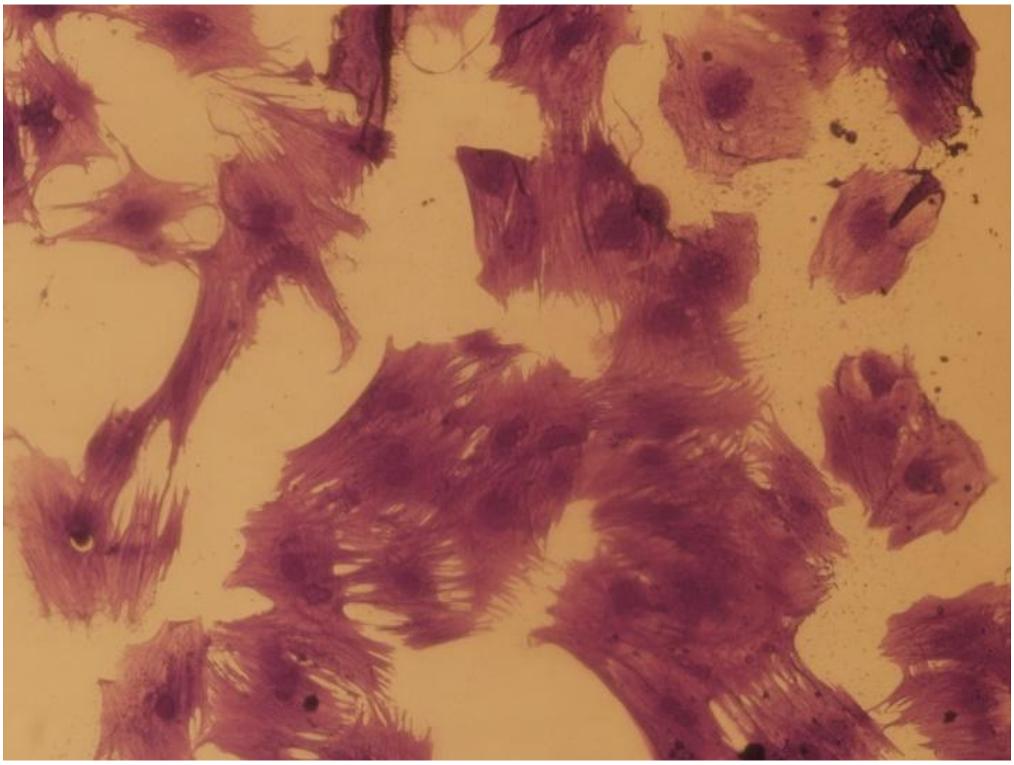

d) Control DFAT cells at 48hours

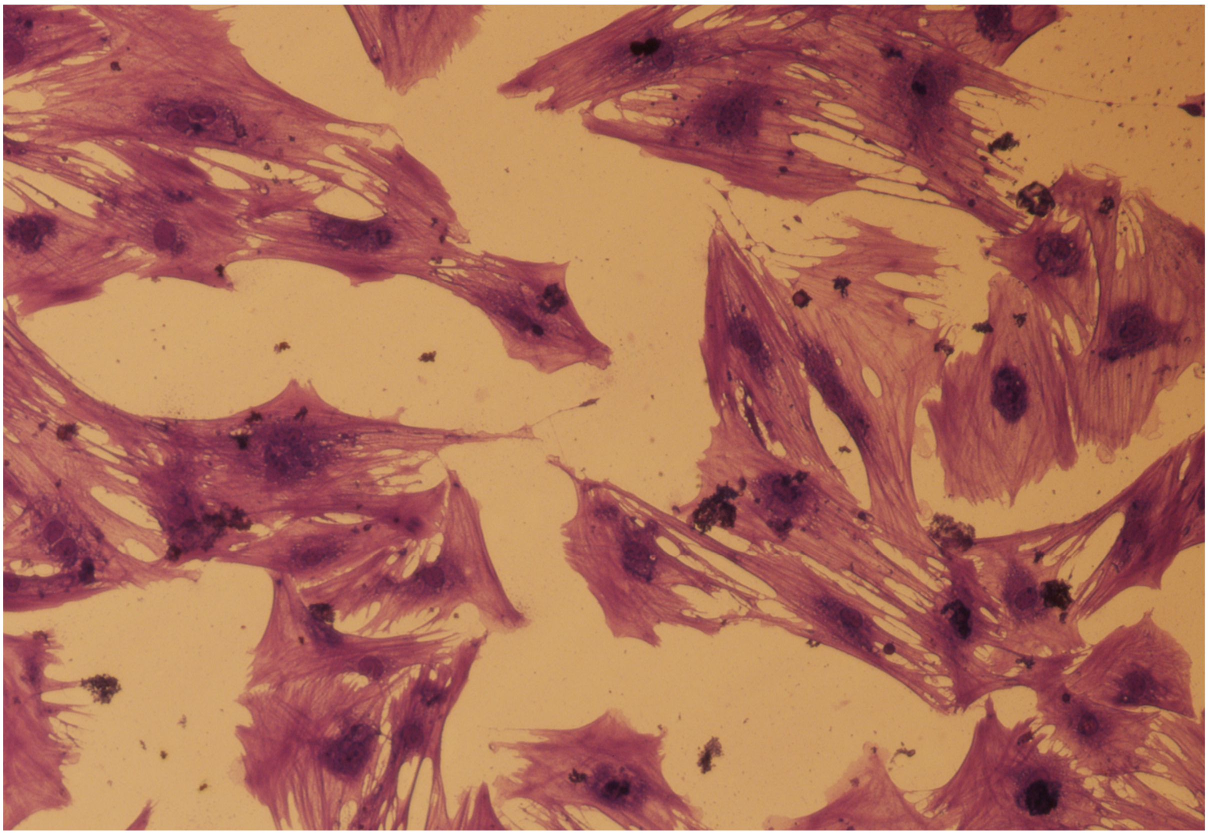

e) 100µM resveratrol treatment of DFAT cells at 48hours

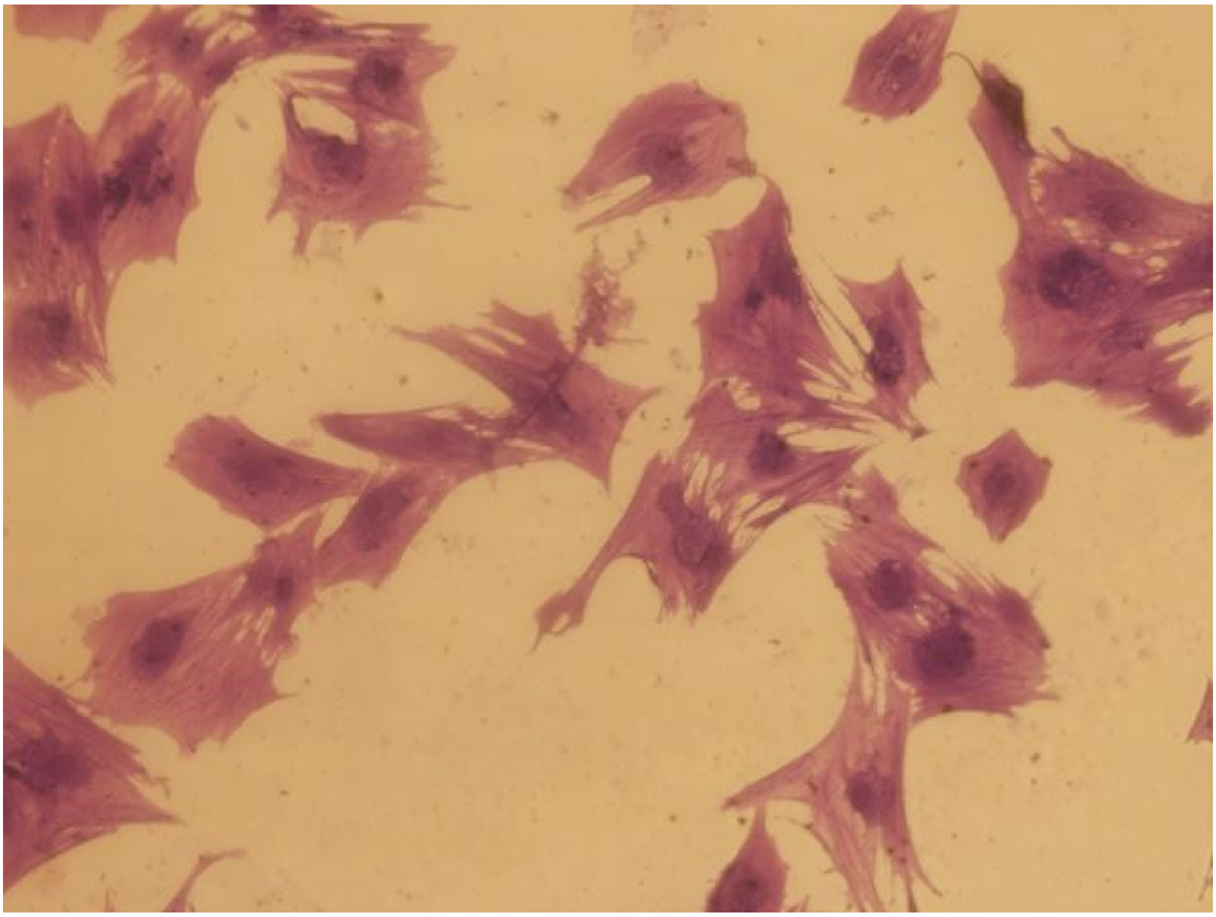

f) 200µM resveratrol treatment of DFAT cells at 48hours

Supplementary Figure S1: Treatment of RSV at different concentrations of DFAT cells

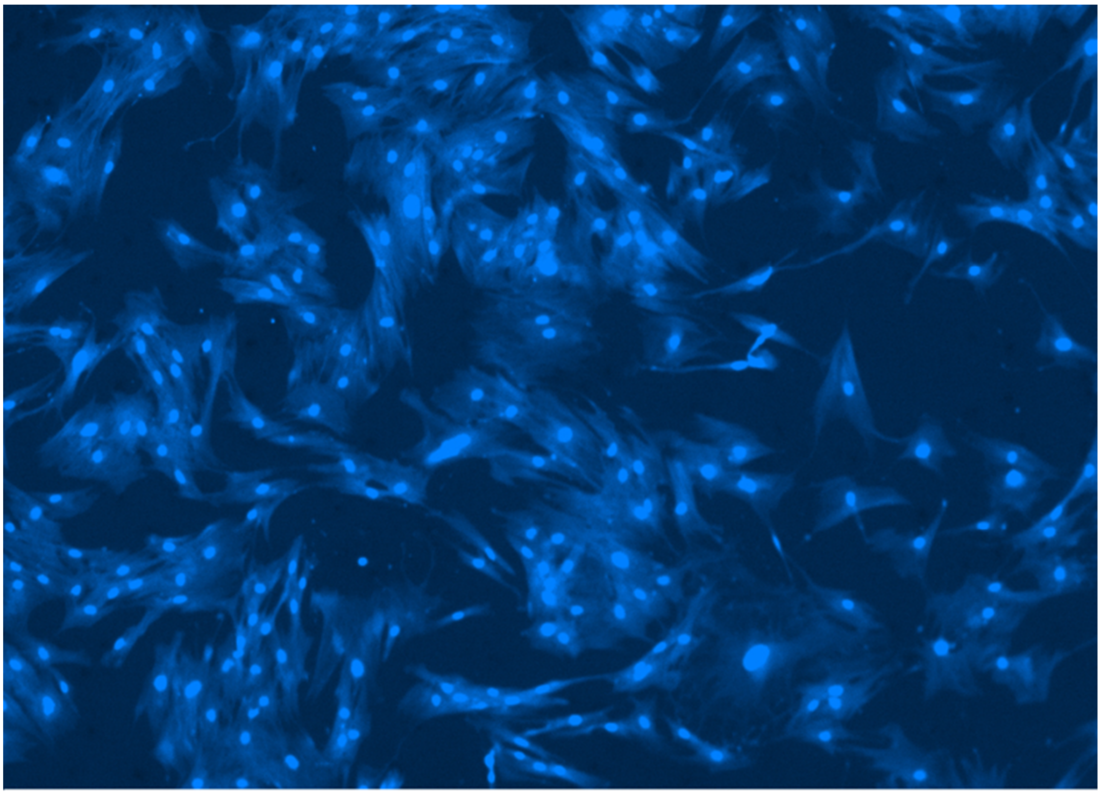

a) DAPI staining of control DFAT cells

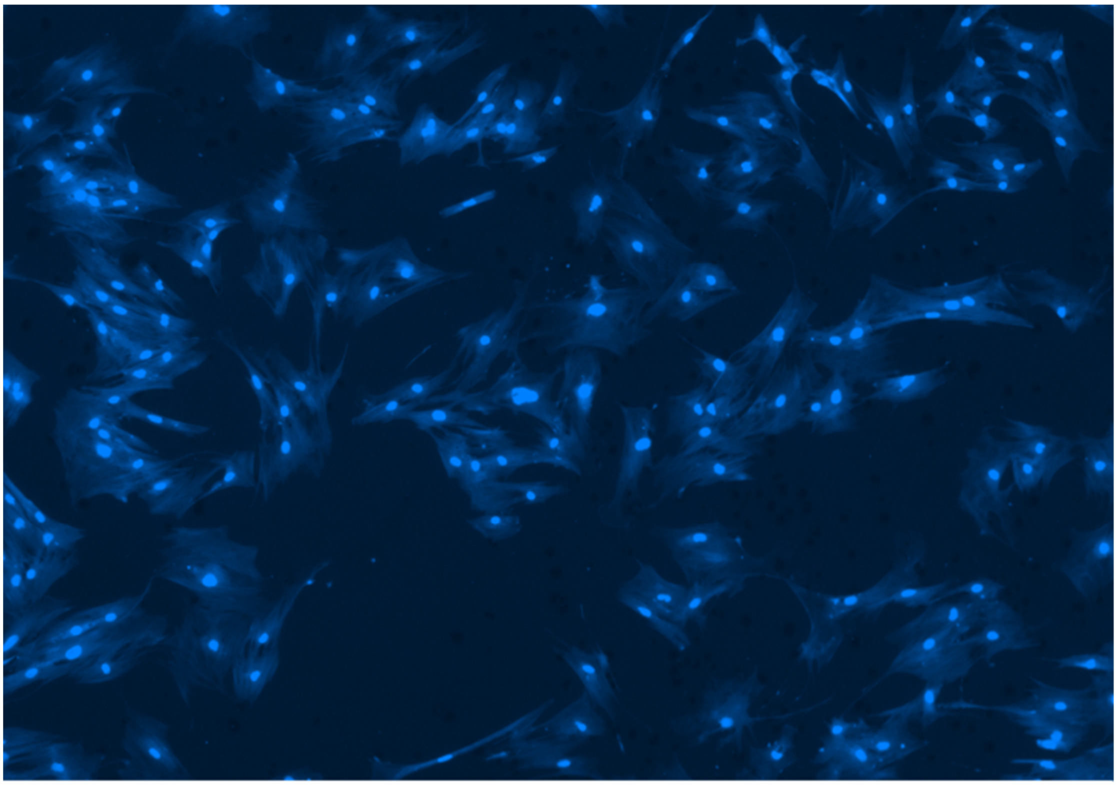

b) DAPI staining of DFAT cells treated with 100µM resveratrol

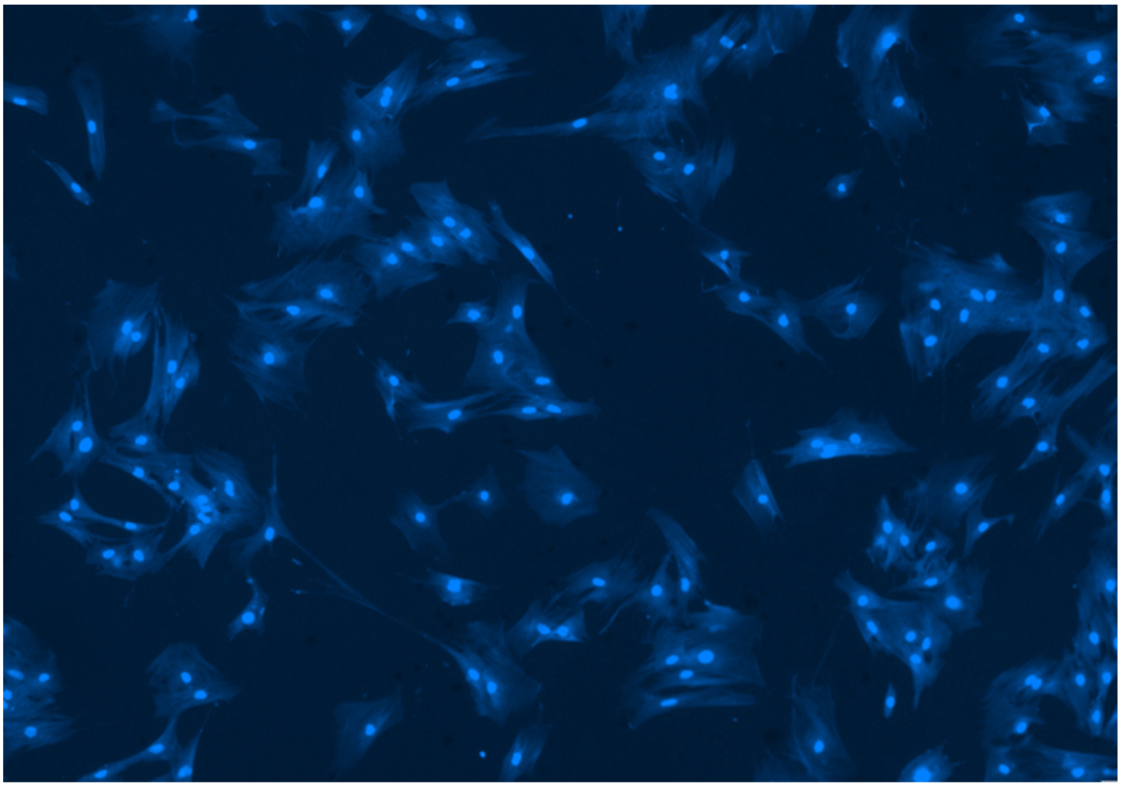

c) DAPI staining of DFAT cells treated with 200µM resveratrol

Supplementary Figure S2: Cell viability of RSV treatment at 100 µM and 200 µM by DAPI staining

Supplementary Table S1: RSV effect on the MDA and ORAC levels in DFAT cells

	Control	RSV treatment		P-value	
		**100µM**	**200µM**	**100µM**	**200µM**
**MDA**	54.569 ± 3.0	43.771 ± 2.1	41.944 ± 2.9	0.01	0.05
**ORAC**	151.982 ± 2.0	155.637 ± 1.9	157.210 ± 2.1	0.008	0.008

Supplementary Table S2: RSV effect on the Relative quantification (RQ) of different genes expression under short and long heat stress

Gene	Isothermal		Short Heat Stress		Long Heat Stress		P-value		
	**Control**	**RSV**	**Control**	**RSV**	**Control**	**RSV**	**HS**	**RSV**	**HS × RSV**
**Oxidative stress**									
STIP1	1.00±0.74	3.93±0.66	1.0±0.72	3.9±0.68	4.17±0.70	7.33±0.73	0.0001	0.0008	0.97
SOD1	1.00±0.29	0.49±0.28	1.11±0.35	0.86±0.29	1.13±0.28	0.550±0.31	0.66	0.21	0.81
HSF1	1.00±0.12	1.02±0.11	0.89±0.12	0.81±0.11	1.09±0.11	0.95±0.12	0.21	0.64	0.74
**Lipid metabolism**									
LIPE	1.00±0.28	1.78±0.27	0.67±0.29	1.51±0.27	0.29±0.26	0.86±0.29	0.009	0.02	0.86
MGLL	1.00±0.21	1.34±0.20	0.80±0.22	0.80±0.20	0.59±0.20	0.62±0.21	0.01	0.60	0.6
PLIN	1.00±0.39	2.44±0.37	0.72±0.41	2.2±0.39	0.88±0.37	2.1±0.41	0.80	0.002	0.93
FASN	1.00±0.09	0.45±0.09	1.03±0.09	0.40±0.09	0.78±0.09	0.42±0.10	0.33	0.0001	0.28
**Apoptosis**									
BAD	1.00±0.13	1.15±0.13	0.88±0.14	1.04±0.13	0.66±0.13	0.96±0.14	0.13	0.20	0.80
BAX	1.00±0.18	2.07±0.18	0.72±0.19	1.72±0.18	1.11±0.18	1.88±0.19	0.1415	0.0001	0.62
PCNA	1.00±0.12	0.44±0.11	0.95±0.12	0.25±0.11	0.57±0.11	0.06±0.12	0.002	0.0001	0.65

**Inflammation**									
CCL2	1.00±0.14	0.36±0.14	0.60±0.15	0.12±0.14	0.88±0.14	0.14±0.15	0.06	0.0006	0.63
IL1-β	1.00±0.53	1.08±0.51	0.72±0.55	0.84±0.53	0.90±0.51	1.04±0.56	0.85	0.85	0.99
**SIRT1 signaling**									
SIRT1	1.00±0.18	0.88±0.18	0.91±0.19	0.71±0.18	0.79±0.17	0.80±0.19	0.65	0.63	0.82
FOXO1	1.00±0.19	0.98±0.18	0.77±0.19	0.77±0.18	0.78±0.18	0.83±0.19	0.39	0.95	0.98
FOXO3	1.00±0.16	0.71±0.17	0.94±0.17	0.52±0.16	1.06±0.16	0.67±0.17	0.62	0.06	0.88
PPARγ	1.00±0.28	1.05±0.26	0.87±0.29	0.65±0.27	0.70±0.26	0.62±0.29	0.34	0.78	0.85
